# Maisonneuve fracture treated with short leg cast: A case report with 41-month follow-up

**DOI:** 10.1097/MD.0000000000038105

**Published:** 2024-05-10

**Authors:** Chaoqun Wang, Shengnan Dong, Xugui Li, Jiakai Ma, Wangcai Wang, Zexi Ling

**Affiliations:** a Traumatic Orthopedic Department, The Affiliated Hospital of Wuhan Sports University, Hongshan District, Wuhan City, Hubei Province, China; b Center of Musculoskeletal Surgery, Charité Medical University Hospital Berlin, Berlin, Germany.

**Keywords:** case report, conservative treatment, inferior tibiofibular syndesmosis, Maisonneuve fracture, short leg cast

## Abstract

**Rationale:**

Maisonneuve fracture is a specific type of severe ankle injury. To our current knowledge, once a Maisonneuve fracture is diagnosed, the surgery is always recommended for fear of sequelae from inaccurate joint reconstruction. However, in this case, we treated a Maisonneuve fracture with a short leg cast, and the 41-month follow-up showed a favorable outcome with no post-traumatic osteoarthritis, chronic pain, and instability. Therefore, this case provides evidence for the feasibility of conservative treatment of Maisonneuve fracture.

**Patient concerns:**

A female patient in her early twenties sprained her left ankle while running, suffering regional pain, swelling, and limited mobility.

**Diagnoses:**

We diagnosed a Maisonneuve fracture with superior fibular fracture and Volkmann tuberosity fracture, a slight separation of inferior tibiofibular syndesmosis (ITS).

**Interventions:**

The patient rejected our surgical recommendations in favor of nonsurgical treatment, in addition to refusing immobilization of the knee. Consequently, we had to treat her with a short leg cast for 8 weeks and asked her to return for regular follow-up visits.

**Outcomes:**

At the final follow-up, the radiography showed complete healing of proximal fibula fracture. The patient reported no discernible subjective differences between her bilateral ankles. The range of motion of the left ankle was measured at 22° of dorsiflexion and 40° of plantarflexion. Functional assessments using Olerud-Molander ankle scale and American Orthopedic Foot and Ankle Society Ankle-Hindfoot scale both scored 100 points. Additionally, the radiographic assessment classified arthritis as stage 0 according to Morrey–Wiedeman classification.

**Lessons:**

To avoid missing and misdiagnosing, the physical examination should always extend to 2 neighboring joints. Secondly, if a Maisonneuve fracture is suspected, further computed tomography scans, radiography, and magnetic resonance imaging can help to determine the stability of the ITS and the integrity of the lateral collateral ligaments before making therapeutic decisions. Finally, considering the lateral collateral ligaments may remain intact, we recommend stabilizing ITS by repairing the medial ligaments, which can be conducted arthroscopically and be more minimally invasive, providing an elastic fixation that aligns better with the biomechanics of the ITS which is characterized as a micro-mobile rather than fully fixed joint.

## 1. Introduction

Maisonneuve fracture is not simply equal to proximal fibular fracture. It is proved to be a special type of severe injury of ankle joint, consisting mainly of the injury of medial malleolus and the fracture of proximal fibula, as well as the rupture of tibiofibular syndesmosis which includes anterior and posterior inferior tibiofibular symphysis and also interosseous membrane of the leg. According to the Lange-Hansen classification, Maisonneuve fracture belongs to the pronation-external rotation type, band 3 or 4. To our knowledge, currently, once a Maisonneuve fracture is diagnosed, the operation is always recommended for fear of sequelae of inaccurate reconstruction of the articulation. However, in this case, we present the treatment of the Maisonneuve fracture with a short leg cast, and the 41-month of follow-up show us a pretty good therapeutic result without the post-traumatic arthritis, chronic pain or instability. As far as we know, there are rare case reports about the conservative treatment for patients with Maisonneuve fractures with a follow-up of more than 36 months, and this is the longest followed case of Maisonneuve fracture treated conservatively.

## 2. Case presentation and surgical technique

The reporting of this study conforms to CARE guidelines.^[1]^ Our institution does not require ethical approval for reporting individual cases or case series. Informed written consent was obtained from the patient for publication of this report and any accompanying images.

A female patient in her early twenties sprained her left ankle by accident while running 2 hours before coming to our outpatient clinic for help. She found it painful in her left ankle and noticed a bit of regional swelling. The pain worsened when she stood on her left foot, so she even could not stand or walk by herself. The condition did not improve after taking a short rest, then she was sent to our outpatient clinic for further treatment. She had no relevant past illness, no history of genetic or familial diseases.

Our first physical examination showed a slight swelling around the patient’s left ankle and tenderness in the anterolateral and posterolateral and anteromedial regions of the ankle. There was no subcutaneous ecchymoses surrounding her ankle, as she had a short interval of 2 hours after the injury. She felt so pain and afraid that she didn’t dare to move her ankle actively in any direction, and when the passive dorsiflexion and plantarflexion happened, the pain worsened. Furthermore, she felt much worse during the passive internal and external rotation of her ankle. We made a mistake that we forgot to examine the superior part of her leg in our first physical examination. However, the second examination was performed after we considered diagnosing her a Maisonneuve fracture. The result showed that she had tenderness and percussion pain in the proximal part of the fibular region.

In the beginning, we only gave her ankle X-rayed examination, it revealed that nothing seemed to be abnormal except for an unsure fracture of posterior malleolus and a slight widening of the inferior tibiofibular space. Immediately after that, we did a computed tomography (CT) scan on her ankle, which showed a Volkmann tuberosity fracture as well as a slight widening of the inferior tibiofibular space, however, without lateral or medial malleolus fractures. The results seemed to violate mechanics rules of rotational ankle injuries, so we began to suspected that she had probably suffered from a Maisonneuve fracture. Then we gave her some additional imaging examinations, including the X-ray of knee, magnetic resonance imaging (MRI) and stress view X-ray of ankle. The results showed a marked proximal fibula fracture, the ruptures of anterior inferior tibiofibular ligament, deltoid ligament and interosseous membrane of her leg. However, stress view radiograph did not show a significant increase in the width of the inferior tibiofibular symphysis compared to non-stress radiograph, in addition, no obvious complete rupture was found with the calcaneofibular ligament and both of the anterior and posterior talofibular ligaments.

Finally, we diagnosed a Maisonneuve fracture with superior fibular fracture and Volkmann tuberosity fracture, a slight separation of inferior tibiofibular symphysis, instable ankle, ruptures of anterior inferior tibiofibular ligament, deltoid ligament and interosseous membrane of the leg.

Due to the potential for post-traumatic arthritis, chronic pain, and instability as sequelae, surgery therapy was strongly recommended for the patient. However, despite our recommendation, the patient expressed reluctance towards surgical intervention and instead opted for a nonsurgical treatment. Nevertheless, she even refused our suggested immobilization of her knee. Consequently, after evaluating the radiography and observing no significant widening changes under the eversion of her left foot, the patient were treated conservatively by a short leg cast for a duration of 8 weeks. Additionally, she was instructed to attend follow-up appointments at our outpatient clinic at designated intervals, namely 1, 2, 4, 6, and 8 weeks following the initial visit.

After 8 weeks of external fixation therapy with a short leg plaster cast, the patient experienced no pain in a resting state. Imaging examination revealed that inferior tibiofibular syndesmosis (ITS) had not further widened compared to the initial measurement. Additionally, signs of healing in the proximal fibula fracture were evident. As a result, at the 8-week follow-up, the plaster cast was removed. However, following the removal of the cast, the patient was instructed not to bear weight on the fractured limb for the first 2 weeks. Instead, she was advised to engage in rehabilitation exercises under non-weight-bearing conditions, including dorsiflexion, plantarflexion, and rotation.

At the 10th week follow-up (2 weeks after removal of the plaster cast), the patient’s left ankle dorsiflexion and plantarflexion had significantly improved, approaching normal levels. However, she continued to experience pain in the anterolateral aspect of her ankle following rehabilitation training. To address this issue, the patient was advised to wear an ankle protector and gradually transition to weight-bearing walking over the next 8 weeks. The weight-bearing regimen involved partial weight-bearing in the initial 4 weeks, followed by total weight-bearing in the subsequent 4 weeks. After the completion of 4 weeks of partial weight-bearing, the patient underwent X-ray and CT scans which indicated union of Volkmann fracture and partial union of the proximal tubular fracture. Following these findings, she was then approved to progress to total weight-bearing walking over the subsequent 4 weeks.

At the 18th week follow-up, the radiography showed complete healing of the proximal fibula fracture. Furthermore, normal dorsiflexion and plantarflexion had recovered, with no claudication remaining, indicating that there was no longer an influence on her normal life and work. However, after total bearing walking for more than 400 m, slight swelling and pain round her ankle would occur.

At the 13th month after the injury, the patient could walk without abnormal gait on the fractured limb under total weight-bearing. There was no discomfort in the left ankle and knee joints at rest, but swelling and pain occurred after low-intensity physical activities, such as playing badminton, jogging, and bicycling, and it was slightly stronger than that of the right side, however, could be significantly relieved after a short period of rest. The range of motion of the left ankle joint spanned from 18° of dorsiflexion to 35° of plantarflexion. The functional assessments revealed a score of 85 points on the Olerud-Molander ankle scale (OMAS) and 85 points on the American Orthopedic Foot and Ankle Society Ankle-Hindfoot scale (AOFAS-AHS). Moreover, the radiological staging for arthritis, according to the Morrey–Wiedeman classification, indicated stage 0.

At the 22nd month after injury, the patient did not experience any pain around the left ankle or left knee while walking under total weight-bearing. Additionally, there was no discernible difference in subjective feeling between the left and right side after engaging in ordinary intensity physical activities. The range of motion of the left ankle was measured at 22° dorsiflexion and 40° plantarflexion. Functional evaluation indicated 100 points in the OMAS and 100 points in the AOFAS-AHS. Furthermore, the radiographic assessment classified arthritis as stage 0 according to Morrey–Wiedeman classification.

**Figure 1.**
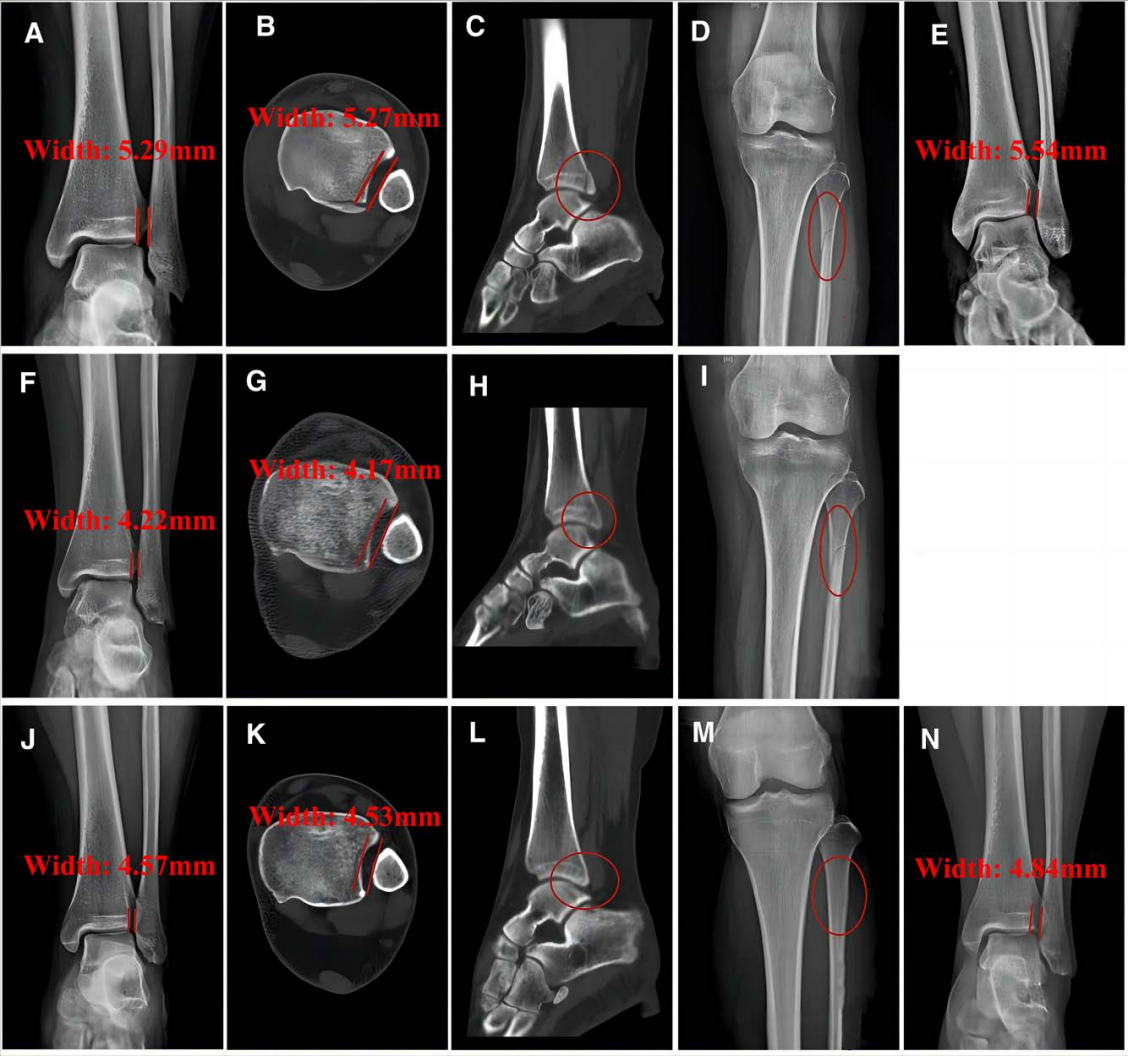
Typical imaging of the patient. (A–E) Radiography and CT scans at the first visit: (A) and (E) represented the non-stress and stress views, respectively; the width of the inferior tibiofibular space was 5.29 mm and 5.54 mm, respectively; (B) showed 5.27 mm in width of inferior tibiofibular space; (C) showed a fracture of the Volkmann tuberosity; (D) showed proximal tubular fracture. (F)–(I) Radiography and CT scans in 14th week after injury: (F) and (G) showed the width of the inferior tibiofibular space was 4.22 mm and 4.17 mm, respectively; (H) revealed the union of Volkmann fracture; (I) showed partial union of proximal tubular fracture, as the fracture line got obscure. (J)–(N) Radiography and CT scans at the 41st month follow-up after injury: (J) and (N) represented that the width of the inferior tibiofibular space on the non-stress and stress views was 4.57 mm and 4.84 mm, respectively; (K) showed 4.53 mm in width of inferior tibiofibular space in a cross section view of CT scans; (L) showed the total union of Volkmann fracture; (M) showed total union of proximal tubular fracture. CT = computed tomography.

At the 41st month after injury, the patient reported no discernible subjective differences between her bilateral ankles. Radiographic images revealed complete healing and well-contouring of the proximal fibular fracture. Further analysis indicated that the width of the ITS, measured on non-stress and stress radiography, was 4.57 mm and 4.84 mm, respectively, with no signs of post-traumatic osteoarthritis or disuse osteoporosis. CT scans of the left ankle demonstrated normal alignment of the ITS, with a measured width of approximately 4.53 mm. The range of motion of the left ankle was measured at 22° of dorsiflexion and 40° of plantarflexion. Functional assessments using OMAS and AOFAS-AHS both scored 100 points. Additionally, the radiographic assessment classified arthritis as stage 0 according to Morrey–Wiedeman classification. For detailed imaging, refer to Figure 1.

## 3. Discussion

Maisonneuve fracture is a special type of severe injury of ankle joint, which is not simply equal to proximal fibular fracture. According to the Lange-Hansen classification, Maisonneuve fracture belongs to the pronation-external rotation type, band 3 or 4. The injury of medial malleolus always occurs first, then follows the lesion of anterior inferior tibiofibular symphysis and interosseous membrane, and finally it goes to the posterior inferior tibiofibular symphysis. In addition, Maisonneuve fracture is characterized on radiograph by a proximal fibula fracture, where the fracture line can even be located near the fibular head.

However, Maisonneuve fracture is highly missed mainly due to the following reasons: A. The fibula fracture line usually locates at the 1/3 superior part of the fibula, but the X-ray and CT scans of ankle cannot show the area; B. Compared to the injury of ankle joint, the pain caused by the superior fibula fracture is slighter, which can be easily ignored by both the orthopedist and the patient, and the patient’s only complaint may be “ankle pain accompanied by the limitation of articular motion,” which cannot easily catch much enough attention from the orthopedist; C. The orthopedist fails to make a comprehensive inquiry, and is not careful enough in the physical examination, probably because he pays all of his attention to the region surrounded by the most pronounced clinical symptoms, ignoring other combined injuries caused by the injury forces transmitted along the axis of our limbs; D. The orthopedist is lack of theoretical knowledge and clinical experience in treating ankle injuries, such as being unaware of the mechanism and the order in which injuries occur. As a result, he only concentrates on the superior fibula fracture and ignores the abnormal widening of the inferior tibiofibular space. In this case, the patient’s chief complaints did not mention the pain around the proximal leg, which did not attract the orthopedist’s attention. Furthermore, the first imaging examination did not involve the 1/3 proximal part of the fibula, the orthopedist did not realized that the patient might suffered from a proximal fibula fracture until CT scans showed the fracture of Volkmann tubercle. Finally the accurate diagnosis was made after the supplementary imaging examinations, thus a missed diagnosis was avoided. However, supposing that the patient in this case had suffered a 3rd degree pronation-external rotation ankle injury according to the Lauge–Hansen classification, that is to say the injury had not yet affected the posterior ITS, and there was no fracture of Volkmann tubercle on the CT scans, then it would have been difficult to avoid a missed diagnosis. Therefore, in order to avoid the missed diagnosis when treating patients suffered from ankle injuries, it is recommended that the scope of the physical examination always extends to the distal and proximal joints near the injured part, considering injury forces can be transmitted along the axis of our limbs.

Most studies^[2–5]^ concluded that when Maisonneuve fracture occurs, ruptures of the interosseous membrane as well as ligaments start at the ITS and end at the level of the proximal fibular fracture, resulting in a complete damage of the ITS, with the stability of the ankle severely impaired, which is also called an extremely unstable ankle fracture. Therefore, once a diagnosis of Maisonneuve fracture confirmed, surgery therapy is strongly recommended for the purpose of recovering the anatomical morphology and accurate alignment of ankle, as well as reconstructing the stability of the ITS, otherwise sequelae such as post-traumatic osteoarthritis and permanent instability of ankle will be easily left.^[5,6]^

However, In order to convince ourselves that the good outcome of this case treated conservatively was not just a coincidence, we reviewed some relevant literatures to search for evidences. In a study by Manyi et al^[7]^ on 12 patients suffered from Maisonneuve fracture, it was proved that the rupture of interosseous membrane extended just from the level of ITS to 32 to 112 mm above the talar dome, which was significantly lower than the level of fibular fracture, and partial remaining interosseous membrane below this level was still intact. In addition, He JQ et al^[8]^ studied the pathoanatomy and injury mechanics of Maisonneuve fracture, the results showed that the rupture of interosseous membrane was located at not only the distal third part of fibula in some cases, but also near the proximal fibula fracture, but the remaining part between these 2 injuried sites was still intact. Therefore, it has been suggested^[9–11]^ that conservative therapy can be recommended for patients suffered from Maisonneuve fracture with interosseous ligament and membrane ruptured while the ITS is still stable, and good prognosis can be achieved even among elite athletes. In our case, the CT scans showed normal morphology of ITS, of which the width measures 5.27 mm (<6.00 mm). In addition, stress X-rays did not show a significant increase in the width of the inferior tibiofibular symphysis compared to non-stress X-rayed views, indicating that the symphysis was still stable, which was one of the reason why our case was suitable for a conservative therapy.

In addition, some literatures^[12–17]^ had also elaborated the connection between lateral ankle stability and lateral collateral ligaments (including mainly calcaneofibular ligament, anterior, and posterior talofibular ligament). They argued that lateral collateral ligaments also contribute a lot to the stability of the distal fibula. In our case, MRI presented that the anterior and posterior talofibular ligaments as well as calcaneofibular ligaments got injured rather ruptured, which confirmed a partial remaining stability of the distal fibula, thus the conservative treatment could be considered. As a result, a 41-month follow-up confirmed a pretty good curative effect without the post-traumatic arthritis, chronic pain or articular instability.

## 4. Conclusion

We present a case that a patient suffering Maisonneuve fracture was treated with a short leg cast instead of surgical treatment, and the 41-month of follow-up showed us a pretty good therapeutic result without the post-traumatic arthritis, chronic pain or instability. To our knowledge, it has been reported that the anterior and posterior talofibular ligaments as well as calcaneofibular ligaments can also contribute partially to the distal regional stability of the fibula,^[12–17]^ and that may be the possible reason why our patient treated with a short leg cast in this case had a good result even in a long time follow-up of more than 3 years. This case can be seen as an evidence that the anterior and posterior talofibular ligament, and the calcaneofibular ligament contribute to the stability of the distal fibula. The lessons we learned from this case are as follows: considering injury forces can be transmitted along the axis of our limbs, the physical examination should be extended to 2 neighboring joints in order to avoid missing and misdiagnosing; once Maisonneuve fracture suspected, additional CT scans, stress X-ray, and MRI can help to determine the stability of the inferior tibiofibular syndesmosis and the integrity of the lateral collateral ligaments, if these structures are found to be stable and integrated, conservative treatment may be recommended; considering the lateral collateral ligament may remain intact, we envision that we can probably try to stabilize the ITS by reconstructing or repairing the medial ligament, which can be conducted arthroscopically, offering a minimally invasive approach that is deemed easier. For instance, we can try to conduct this procedure by using suturing anchors, which provide an elastic fixation that aligns better with the biomechanics of the ITS compared to internal fixation with screws. Given that the ITS is characterized as a micro-mobile rather than fully fixed joint, this method may offer improved outcomes.

However, this study is only a report of a particular case rather than a clinical study of a large sample, which is the major limitation of our study. In addition, during the follow-up, we did not continue to perform MRI on the patient due to the nonroutine nature of MRI, which resulted in our inability to provide adequate evidence related to the ligament injury and repair. Therefore, we emphasis that further biomechanical studies should be conducted to validate the specific influence of intact lateral collateral ligaments, such as the calcaneofibular ligament and anterior and posterior talofibular ligaments, on maintaining the stability of the ITS after a severe injury, and our research team is committed to conducting these experiments in future investigations, with particular emphasis on evaluating the extent of lateral collateral ligament damage in Maisonneuve fracture patients. Subsequently, we intend to explore the potential of using medial ligament repair as a novel therapeutic avenue for treating Maisonneuve fractures in cases where the lateral collateral ligament remains intact. This focus on innovative surgical techniques may provide new directions for scientific research and may even ultimately redefine current surgical methods and principles in the management of Maisonneuve fractures.

## Acknowledgments

We would like to thank the nursing team of Traumatic Orthopedic Outpatient Clinic of the Affiliated Hospital of Wuhan Sports University, for the support. This study was supported by China Scholarship Council (No. 202308420035).

## Author contributions

**Conceptualization:** Chaoqun Wang, Wangcai Wang, Zexi Ling.

**Funding acquisition:** Chaoqun Wang.

**Project administration:** Zexi Ling.

**Resources:** Chaoqun Wang, Jiakai Ma, Wangcai Wang.

**Supervision:** Zexi Ling.

**Visualization:** Chaoqun Wang, Jiakai Ma.

**Writing – original draft:** Chaoqun Wang, Shengnan Dong.

**Writing – review & editing:** Chaoqun Wang, Shengnan Dong, Xugui Li.
